# The *Pseudomonas aeruginosa* Secreted Protein PA3611 Promotes Bronchial Epithelial Cell Epithelial-Mesenchymal Transition via Integrin αvβ6-Mediated TGF-β1-Induced p38/NF-κB Pathway Activation

**DOI:** 10.3389/fmicb.2021.763749

**Published:** 2022-02-07

**Authors:** Lei Shu, Sixia Chen, Shaoqing Lin, Huan Lin, Yan Shao, Jing Yao, Lili Qu, Yunshi Zhang, Xing Liu, Xingran Du, Kaili Deng, Xiaolin Chen, Ganzhu Feng

**Affiliations:** ^1^Department of Pulmonary and Critical Care Medicine, The Second Affiliated Hospital of Nanjing Medical University, Nanjing, China; ^2^Department of Respiratory Medicine, Sir Run Run Shaw Hospital, Nanjing Medical University, Nanjing, China; ^3^Tongji Hospital, Tongji University School of Medicine, Tongji University, Shanghai, China; ^4^Jiangsu Provincial Center for Disease Control and Prevention, Nanjing, China; ^5^Laboratory Medicine Center, The Second Affiliated Hospital, Nanjing Medical University, Nanjing, China; ^6^Department of Tuberculosis, Xuzhou Infectious Disease Hospital, Xuzhou, China; ^7^Department of Respiratory Medicine, The Affiliated Suqian Hospital of Xuzhou Medical University, Suqian, China; ^8^Department of Infectious Diseases, The Second Affiliated Hospital of Nanjing Medical University, Nanjing, China

**Keywords:** *Pseudomonas aeruginosa*, bronchial epithelial cells, PA3611, integrin αvβ6, epithelial-mesenchymal transition

## Abstract

*Pseudomonas aeruginosa* (PA) is an important pathogen that has been proven to colonize and cause infection in the respiratory tract of patients with structural lung diseases and to lead to bronchial fibrosis. The development of pulmonary fibrosis is a complication of PA colonization of the airway, resulting from repeated infection, damage and repair of the epithelium. Bronchial epithelial cell epithelial-mesenchymal transition (EMT) plays a vital role in bronchial fibrosis. To date, research on bronchial epithelial cell EMT caused by PA-secreted virulence factors has not been reported. Here, we found that PA3611 protein stimulation induced bronchial epithelial cell EMT with mesenchymal cell marker upregulation and epithelial cell marker downregulation. Moreover, integrin αvβ6 expression and TGF-β1 secretion were markedly increased, and p38 MAPK phosphorylation and NF-κB p65 subunit phosphorylation were markedly enhanced. Further research revealed that PA3611 promoted EMT via integrin αvβ6-mediated TGF-β1-induced p38/NF-κB pathway activation. The function of PA3611 was also verified in PA-infected rats, and the results showed that ΔPA3611 reduced lung inflammation and EMT. Overall, our results revealed that PA3611 promoted EMT via integrin αvβ6-mediated TGF-β1-induced p38/NF-κB pathway activation, suggesting that PA3611 acts as a crucial virulence factor in bronchial epithelial cell EMT and is a potential target for the clinical treatment of bronchial EMT and fibrosis caused by chronic PA infection.

## Graphical Abstract

The role of PA3611 on EMT.

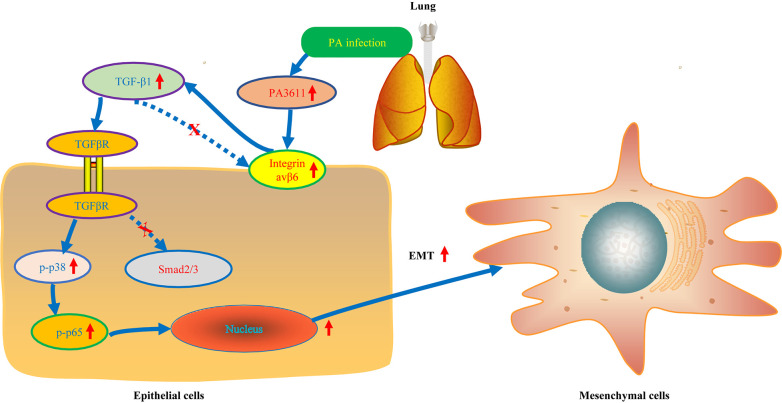

## Introduction

*Pseudomonas aeruginosa* (PA) is a type of non-fermenting gram-negative bacterium that can cause various acute and chronic infections, including infections of the blood system, urinary system, central nervous system, bone and joints. PA is also one of the most common pathogens that cause hospital-acquired infections, including pneumonia (Li et al., 2016), and is an opportunistic pathogen and the main cause of morbidity and death in cystic fibrosis patients and immunocompromised individuals (Pang et al., 2019). As an opportunistic bacterium, PA can cause acute and chronic infection throughout the body. However, it plays a particularly important role in chronic respiratory infections of patients who already have a lung condition or a dysregulated immune system. Importantly, it does not respond to commonly used antibiotics, which means that infections can be difficult to treat (Williams et al., 2010; Curran et al., 2018; Garcia-Clemente et al., 2020). Therefore, it is an urgent and important task to actively seek effective countermeasures against PA infection. PA is widespread in nature, prone to translocation, and likely to cause infection of the respiratory tract, especially in patients with structural lung diseases, such as chronic obstructive pulmonary disease (COPD), bronchiectasis, and cystic fibrosis (Gomez-Junyent et al., 2014; Boixeda et al., 2015). PA infection may promote the formation of bronchial fibrosis, and the development of pulmonary fibrosis is a complication of PA colonization of the airway, resulting from repeated infection, damage and repair of the epithelium, including thickening of the airway wall and lumen stenosis, in patients with COPD, bronchiectasis and cystic fibrosis, thereby aggravating the irreversible airflow limitation of patients (de Bentzmann et al., 1996; Losa et al., 2015; Ruffin and Brochiero, 2019). Studies have shown that the pathological manifestations of bronchial fibrosis are mainly airway smooth muscle thickening, extracellular matrix deposition, myofibroblast proliferation and transition of airway epithelial cells into mesenchymal cells (epithelial-mesenchymal transition, EMT) (Grzela et al., 2016). Additionally, lung-resident mesenchymal stem cells can differentiate into myofibroblasts activated by inflammatory M2 macrophages (Hou et al., 2018), and inflammatory macrophages may transdifferentiate into myofibroblasts (Meng et al., 2016). Among these manifestations, EMT plays a key role in the process of bronchial fibrosis (Nowrin et al., 2014), which involves cells losing their original polarity, transforming into mesenchymal cells and exhibiting characteristics of pathological processes. However, it is unclear whether the changes in ultrastructure are related to the inhibition of cell proliferation. The expression of original epithelial cell markers, such as *E*-cadherin and tight junction protein 1 (zonula occludens-1, ZO-1), is reduced, while mesenchymal cell markers, such as α-smooth muscle actin (α-SMA), *N*-cadherin (*N*-cad) and vimentin, are increased, and the activity of some matrix metalloproteinases (MMPs) is also increased. Among these features, high expression of α-SMA, vimentin and MMP-9 can be used as important signs of EMT (Meng et al., 2015).

In recent years, although EMT studies on airway epithelial cells in patients with COPD have been reported, most of them have used cigarette smoke-induced COPD models as the main research subject or used only the total lytic substance of PA bacteria as the cell irritant (Borthwick et al., 2011; Milara et al., 2013; Wang et al., 2013). Studies on the EMT of bronchial epithelial cells caused by the secretion of virulence factors from chronic PA infections have not been reported.

PA3611 is a toxic protein secreted during PA infection (UniProt ID: Q9HY15). Its encoding gene is 411 base pairs in length (including the signal peptide), and its molecular weight is 14.986 kDa. Proteomic analysis has shown that PA3611 may be a virulence factor regulated by the quorum sensing system (Nouwens et al., 2003). Some preliminary studies have shown that the spatial structure of PA3611 is composed of five β-chains (B1-B5) and five α-helix (H1-H5) domains (Das et al., 2014); however, its specific biological functions and its related effects on infected host cells have not yet been reported.

In this study, we elucidated the role and related mechanism of PA3611 secreted by PA in the process of inducing bronchial epithelial cell EMT and provided a new potential therapeutic target for the clinical treatment of chronic PA infection leading to bronchial EMT and fibrosis.

## Materials and Methods

### Recombinant PA3611 Protein *in vitro*

A DNA fragment encoding PA3611 was obtained from Qiangyao Biological Company (Suzhou, China), and oligonucleotide primers were designed with Primer 5.0 (Premier, Canada) (Supplementary Table S1). The DNA template and primers were synthesized by Qiangyao, and PCR was performed with the template described above. The PCR amplification conditions were as follows: 94°C for 5 min, followed by 30 cycles of 96°C for 25 s, 58°C for 25 s, and 72°C for 1 min. The PCR product was cut and purified via a DNA gel extraction kit (Axygen, NY, United States). The purified PCR product and pET-28a(+) vector (Novagen, Germany) were digested with *Bam*HI and *Xho*I (NEB) (Thermo Scientific, DE, United States) and ligated together with T4 DNA ligase (Thermo Scientific). The pET-28a(+) vector containing the PA3611-encoding gene was transformed into *Escherichia coli* strain BL21 (DE3) (Novagen), and the cells were subsequently grown on Luria-Bertani (LB) agar plates containing 50 μg/mL kanamycin at 37°C overnight. Positive clones were selected for enzyme digestion and sequencing identification. The sequence-corrected plasmids were transformed into *E. coli* BL21 (DE3), which were plated onto solid medium containing kanamycin and incubated overnight at 37°C at 250 r/min. One percent of overnight bacteria were transferred to LB medium containing kanamycin and incubated at 37°C at 250 r/min for 3 h, 0.5 mmol/L IPTG was added, and the cells were incubated at 20°C for 12 h. Next, the culture was centrifuged, and the cells were resuspended and lysed by sonication. The sonicated sample was centrifuged, and the supernatant of the cell lysate was applied to Ni-IDA resin (Qiangyao). The protein was subsequently eluted and collected for SDS-polyacrylamide gel electrophoresis (SDS–PAGE) analysis. The concentration of the protein was determined by the Bradford method. Subsequently, Western blotting was performed to examine the purified recombinant PA3611 protein via N-His Tag (His Tag antibody; Qiangyao) analysis. Then, the purified protein was filtered and sterilized with a 0.22-μm sterile membrane and stored at –80°C until use.

### Cells and Bacterial Culture

The human bronchial epithelial cell line 16HBE14o-, which was originally created by Prof. D. Gruenert (Cozens et al., 1994), and the rat bronchial epithelial cell line RTE were purchased from Procell Company (Wuhan, China) and preserved in our laboratory. Cells were cultured in RPMI-1640 medium (16HBE14o-) or low glucose DMEM (RTE) (Gibco BRL, Grand Island, NY, United States) containing 10% fetal bovine serum (FBS, Gibco BRL), penicillin (100 U/mL), streptomycin (100 μg/mL), and L-glutamine (2 mM) and placed in a cell incubator at a constant temperature of 37°C and 5% CO_2_ under a humidified atmosphere. The medium was changed every other day, the cells were passaged when approximately 80% cell confluency was achieved, and passages 3–8 cells were used for experimentation.

The PA standard strain PAO1 (ATCC15692) was kindly donated by Professor Jinfu Xu from Shanghai Chest Hospital. The PA knockout strain ΔPA3611 was purchased from Guangzhou Nuojing Biotechnology Company, and both strains were cultivated on cetrimide-agar (Merck, Darmstadt, Germany) plates. For the experiments, single colonies of bacteria were inoculated in LB broth (Merck), incubated overnight at 37°C and 140 rpm and stored at –80°C. Before infecting animals, the bacteria were resuscitated and grown to reach exponential phase in TSB with 0.5% arabinose. Next, bacterial cells were pelleted by centrifugation and washed twice with sterile PBS and the OD of the bacterial suspension was adjusted by spectrophotometry at 600 nm.

### Cell Transfection Assay

The fragments encoding the p65 and p38 gene alleles were obtained from GenScript and cloned into pcDNA3.1 (Invitrogen, Carlsbad, CA, United States). The cell lines 16HBE14o- and RTE were transiently transfected with pcDNA3.1/p65 cDNA, pcDNA3.1/p38 cDNA, and control pcDNA3.1 using Lipofectamine 3000 (Invitrogen) according to the manufacturer’s instructions. Cells were incubated for 24 h at 37°C before being used for further analysis.

Small interfering RNAs (siRNAs) targeting NF-κB p65 subunit mRNA or p38 mRNA and a random non-coding siRNA were synthesized by GenePharma. 16HBE14o- and RTE cells were transfected with the above siRNAs using Lipofectamine 3000 according to the manufacturer’s instructions. The non-coding siRNA was used as a normal control (NC). The cells were transfected with the above RNAs for 24 h and then administered PBS (negative control) or PA3611 (10 μg/mL) for another 24 h, and further experiments were performed. The corresponding sequences of these RNAs are shown in Supplementary Table S2.

### Proliferation Assay

16HBE14o- and RTE cells were seeded in a 96-well cell culture plate at 6,000 cells per well and cultured overnight. Then, PA3611 was added at protein concentrations of 0, 1, 5, 10, and 50 μg/mL, and RPMI-1640 or low-glucose DMEM with 10% FBS was used to adjust the total volume of each well to 100 μL (the culture solution was changed every 24 h, and the PA3611 protein concentration was kept consistent). After incubation for 24, 48, or 72 h, the proliferation assay was performed using a CCK-8 kit (Dojindo Laboratories, Kumamoto, Japan) according to the manufacturer’s protocol.

### Enzyme-Linked Immunosorbent Assay

To determine the secretion of active TGF-β1, cell-free supernatants were collected and used to evaluate the concentrations of TGF-β1 with human and rat TGF-β1 Enzyme-Linked Immunosorbent Assay (ELISA) kits (R&D Systems, Minneapolis, MN, United States) according to the manufacturer’s instructions.

### Western Blotting Analyses

16HBE14o- and RTE cells incubated for different time periods were collected and lysed. The lysates were centrifuged, denatured, applied to SDS polyacrylamide gels for electrophoresis and transferred to polyvinylidene fluoride membranes. Then, the membranes were blocked with 5% skimmed milk at room temperature for 1 h, and immunoblotting was performed using antibodies against NF-κB p65 (Abcam, MA, United States), NF-κB p-p65 (phospho S536, Abcam), p38 (Abcam), p-p38 (phospho Y182, Abcam), α-SMA (Abcam), vimentin (Abcam), *E*-cadherin (Abcam), ZO-1 (Abcam), Smad2/3 (Abcam), p-Smad2/3 (Abcam), integrin avβ6 (Abcam), β-actin (Abcam) and a TGF-β1-neutralizing antibody (Sigma). After washing, the membranes were incubated with the appropriate horseradish peroxidase-conjugated secondary antibody at room temperature for 1 h. The bands were visualized via chemiluminescence using an ECL kit (Thermo Scientific) and photographed with a Tanon Multi-Imager. ImageJ (Scion Corporation, Frederick, MD, United States) was used to measure the density of the immunoreactive bands, which was normalized to that of β-actin.

### Quantitative Reverse Transcriptase-PCR

Total RNA was extracted from the cells using a Total RNA Extraction Kit (Generay Biotech, Shanghai, China), RNA was reverse-transcribed into cDNA using a RevertAid First Strand cDNA synthesis Kit (Thermo Scientific Fisher, Waltham, MA, United States), and the thermocycling program used was as follows: 37°C for 60 min and 85°C for 5 min. Amplifications were performed in an iCycler using iQ SYBR Green Supermix (Bio-Rad). GAPDH was amplified on the same plates and used to normalize the data. Each sample was prepared in triplicate, and each experiment was repeated at least three times. The relative abundance of each gene was quantified using the 2^–ΔΔCt^ method. The PCR primers used are listed in Supplementary Table S1.

### Preparation of Agarose-Coated Bacteria

A 2% agarose solution of 100 mL (in PBS) was prepared. Tryptic soy broth (TSB) (3.0 g) was added to 100 mL of distilled water and autoclaved at 121°C for 15 min. PA strains (PAO1 and ΔPA3611) were inoculated into solid medium and incubated overnight at 37°C. Then, selected colonies were inoculated into 5 mL of TSB and cultured overnight. One milliliter of the overnight culture was put into a flask containing 10 mL of TSB and incubated at 37°C and 250 rpm until the logarithmic phase. Then, the bacterial culture was added to 10 mL of preheated (48°C) agarose solution. The agarose bacterial solution was mixed quickly by vortexing, poured into preheated paraffin oil and stirred at 500 rpm for 5 min. The excess paraffin oil was cooled and removed with a vacuum pump and stored at 4°C for use.

### Infection of Rats *in vivo*

Six- to eight-week-old male Wistar rats (Cavens Lab Animal Company, Changzhou, China) were housed in a biosafety level III animal facility under specific pathogen-free conditions. All animal experimental procedures were approved by the Institutional Animal Ethics Committee of the Second Affiliated Hospital of Nanjing Medical University (No. 2014KY050) and were performed in strict accordance with Nanjing Medical University’s guidelines for the use of laboratory animals. All rats were divided into four groups: the control group (PBS group), agarose group, agarose-coated PAO1 group (PAO1 group) and agarose-coated ΔPA3611 group (△PA3611 group). Lung tissues were collected for histological and immunohistochemical staining at week 2, week 4, and week 6 after intratracheal injection of agarose-coated suspension or 120 μl of PBS every 2 weeks for a total of three injections.

### Histological and Immunohistochemical Staining

Lung lobes were collected and fixed with 4% paraformaldehyde overnight and embedded in paraffin. Hematoxylin and eosin (HE)-stained tissues were assessed via pathological analysis. Lung injury was estimated by the percentage of the lesion area in the total lung area using an ImagePro macro. Immunohistochemical staining of α-SMA, vimentin, *E*-cadherin, ZO-1 and integrin αvβ6 was performed using tissue sections that were dewaxed and rehydrated. Antigen retrieval was performed using proteinase K and hot citric acid buffer treatment as needed. The restored sections were incubated with primary antibodies overnight at 4°C. After rinsing with Tris-buffered saline for 15 min, sections were incubated with secondary antibody (biotinylated goat anti-rabbit IgG, Sigma). Sections were then washed and incubated with Vectastain Elite ABC reagent (Vector Laboratories, Burlington, ON, Canada) for 45 min. Staining was developed using 3,3-diaminobenzidine (2.5 mg/mL) followed by counterstaining with Mayer’s hematoxylin. Images were taken with a Leica Microsystems Ltd. microscope and subsequently analyzed independently with Image-Pro Plus 6 by two pathologists who had no experimental information.

### Immunofluorescence Staining

16HBE14o- and RTE cells were cultured on chamber slides (Lab-Tek II Chamber Slide system, Naperville, IL, United States) with PBS, PA3611 (10 μg/mL), TGF-β1 (2 ng/mL), or PA3611 (10 μg/mL) + TGF-β1-neutralizing antibody (10 μg/mL) for 48 h at 37°C. Cells were then fixed with 10% formalin, permeabilized with acetone and stained with rabbit anti-α-SMA, -vimentin, -*E*-cadherin, -ZO-1 or -integrin αvβ6 antibodies followed by Alexa Fluor-488-goat anti-rabbit or Alexa Fluor-568-goat anti-rabbit secondary antibodies (Invitrogen). The images were taken at 100 × magnification using a confocal microscope (FV1000 Olympus).

### Bacterial Load Assessment in the Lung

To determine the bacterial loads in the lungs, the rats were killed at 2, 4, or 6 weeks after infection. The lungs were removed aseptically, weighed, and homogenized. Serial dilutions of tissue homogenates were plated onto Muller-Hinton II agar plates using serial dilutions, and bacterial colony-forming units (CFUs) were enumerated after incubation at 37°C for 18 h.

### Transmission Electron Microscopy Examination

16HBE14o- and RTE cells were rinsed with ice-cold 0.1 M PBS (pH 7.4) and centrifuged at 500 × *g* for 5 min at room temperature, after which the clear supernatants were removed. Cell pellets were fixed with 2.5% glutaraldehyde for at least 30 min at 4°C. After fixation, the treated cells were thoroughly washed in PBS and then postfixed with 1% OsO4 for 1 h at room temperature. Then, the specimens were embedded in Epon for 12 h at 35°C. Finally, 50–70-nm sections were stained with uranyl acetate (30 min) and lead citrate (10 min) at room temperature. Images were observed using a JEM-2000EX transmission electron microscope at 60 kV.

### Statistical Analysis

All the presented results are expressed as the mean ± SD and were confirmed in three independent experiments. Student’s *t*-test was used to compare two groups, and multiple groups were analyzed by one-way ANOVA. Statistical analyses were performed using SPSS 20.0 (IBM SPSS, Armonk, NY, United States). *P* < 0.05 was considered to indicate a significant difference.

## Results

### Heterologous Expression and Purification of Recombinant PA3611

The PA3611 protein was synthesized and purified for further investigation. The PA3611-encoding gene was amplified by PCR, cloned into the plasmid pET-28a(+), and then transformed into *E. coli* BL21 (DE3). The positive clone and recombinant protein were confirmed by sequencing and SDS-PAGE, and the results are shown in Supplementary Figures 1A,B. The recombinant PA3611 protein was expressed in *E. coli* BL21 (DE3) and was subsequently purified with Ni-IDA resin, and endotoxin was removed with ultrafiltration.

### PA3611 Inhibited the Proliferation of Bronchial Epithelial Cells and Promoted the Transformation of Epithelial Cells Into Mesenchymal Cells

**FIGURE 1:**
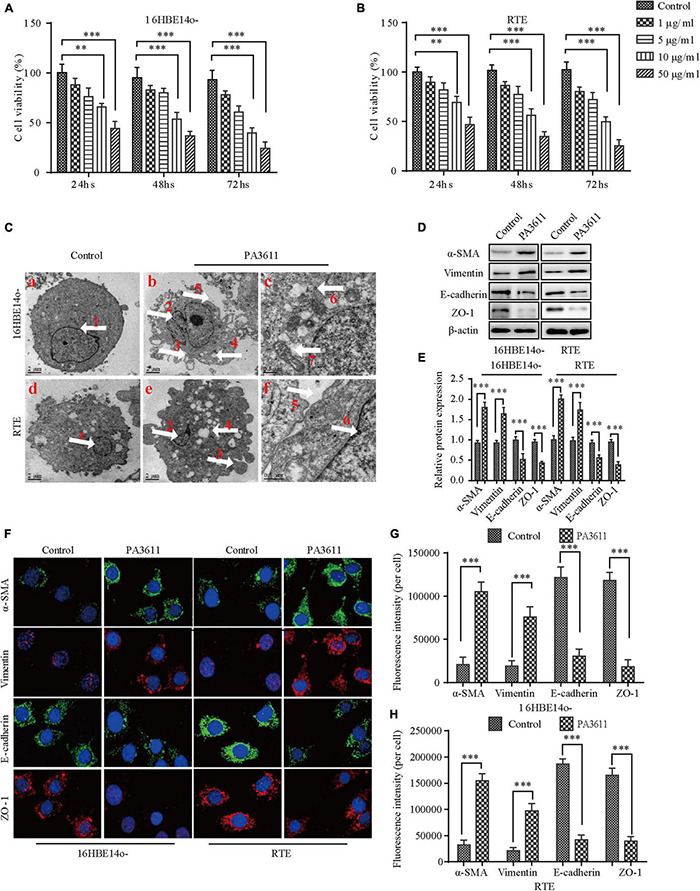
Effects of PA3611 on the proliferation and epithelial-mesenchymal transition (EMT) of bronchial epithelial cells. 16HBE14o- and RTE cells were treated with different amounts of PA3611 (1, 5, 10, and 50 μg) for 24, 48, or 72 h. A CCK-8 assay was performed to estimate cell proliferation **(A,B)**. Transmission electron microscopy was used to observe the ultrastructure of the above cells treated with PA3611 (10 μg/mL) for 48 h **(C)**. EMT-related protein (α-SMA, vimentin, *E*-cadherin and ZO-1) levels in 16HBE14o- and RTE cells at 48 h were assessed by Western blot analysis **(D–F)**. Fluorescence intensities of EMT-related proteins (α-SMA, vimentin, *E*-cadherin and ZO-1) were assessed by immunofluorescence staining in 16HBE14o- and RTE cells at 48 h **(F–H)**, and photographs were obtained with a confocal laser microscope (100 × objective lens). All the presented graphs are representative of the results of three independent experiments. The data are presented as the mean ± SD. *P < 0.05, ^**^P < 0.01, ^***^P < 0.001, vs. the control group. White arrow ① and ② nucleus; ③ cells crest; ④ cells vacuolation; ⑤ intercellular gaps; ⑥ intracellular glycogen; ⑦ mitochondria.

To explore the function of PA3611 in bronchial epithelial cells, a cell counting kit-8 (CCK-8) assay was used to estimate the effect of PA3611 on bronchial epithelial cell proliferation. The results demonstrated that PA3611 significantly inhibited 16HBE14o- and RTE cell proliferation, and this effect was increased with higher concentrations of PA3611 and longer incubation times (Figures 1A,B), indicating that PA3611 inhibited cell proliferation in a time- and dose-dependent manner.

To further understand whether the internal ultrastructure of the above two bronchial epithelial cells was changed after stimulation with the PA3611 protein, Transmission Electron Microscopy (TEM) was used to observe the ultrastructure of the above cells treated with PA3611 (10 μg/mL) for 48 h. The results showed that intercellular gaps were widened, intercellular connections disappeared, filamentous pseudopodia appeared, nuclei were heteromorphic, perinuclear gaps were widened, mitochondria were swollen and distorted, and intracellular glycogen was increased, as shown in Figure 1C. These results indicated that the PA3611 protein could promote the transformation of bronchial epithelial cells into mesenchymal cells at a certain concentration and after a specific amount of time.

To verify that the above two bronchial epithelial cells underwent EMT after stimulation with the PA3611 protein, EMT-related marker (α-SMA, vimentin, *E*-cadherin and ZO-1) expression was assessed by Western blot analysis and immunofluorescence staining. The results showed that PA3611 significantly increased α-SMA and vimentin expression and decreased *E*-cadherin and ZO-1 protein expression (Figures 1D,E). Similar results were also shown by immunofluorescence staining; the fluorescence intensities of α-SMA and vimentin were significantly increased, and those of *E*-cadherin and ZO-1 were significantly decreased after treatment with PA3611 (10 μg/mL) for 48 h (Figures 1F–H). These results indicated that PA3611 can stimulate bronchial epithelial cell EMT *in vitro*.

### PA3611-Stimulated TGF-β1 Production Was Accompanied by the Promotion of Bronchial Epithelial Cell Epithelial-Mesenchymal Transition and p65 Phosphorylation

**FIGURE 2:**
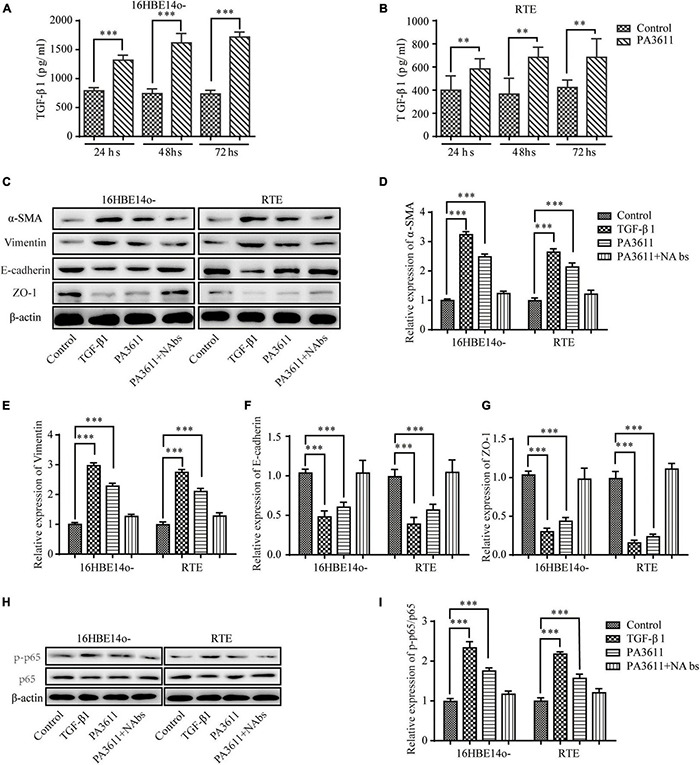
Effects of PA3611 and TGF-β1 on the expression of p65 phosphorylation and EMT-related markers. 16HBE14o- and RTE cells were treated with PA3611 (10 μg/mL), and the TGF-β1 levels in 16HBE14o- and RTE cell culture supernatants were detected by an enzyme-linked immunosorbent assay (ELISA) at 24, 48, and 72 h **(A,B)**. 16HBE14o- and RTE cells were treated with PA3611 (10 μg), TGF-β1 (2 ng/mL) or PA3611 (10 μg) plus TGF-β1-neutralizing antibody (10 μg/mL) for 48 h, and EMT-related markers (α-SMA, vimentin, *E*-cadherin and ZO-1, **C–G**) and p65 phosphorylation **(H,I)** were then assessed by Western blot analysis. All the presented graphs are representative of the results of three independent experiments. The data are presented as the mean ± SD. *P < 0.05, ^**^P < 0.01, ^***^P < 0.001, vs. the control group.

TGF-β1 is a key cytokine and participates in the EMT process (Qian et al., 2018; Sun et al., 2018). To determine whether PA3611 promoted bronchial epithelial cell EMT by stimulating TGF-β1 production, the TGF-β1 levels in 16HBE14o- and RTE cell culture supernatants were evaluated by ELISA at 24-, 48-, and 72-h time points. The results showed that the TGF-β1 levels in supernatants were increased after PA3611 stimulation in both 16HBE14o- and RTE cells at all three time points (Figures 2A,B).

To further confirm that PA3611-promoted bronchial epithelial cell EMT was regulated by TGF-β1, 16HBE14o- and RTE cells were treated with PBS (negative control), TGF-β1 (2 ng/mL, positive control), PA3611 (10 μg/mL), and PA3611 (10 μg/mL) + TGF-β1-neutralizing antibody (10 μg/mL) for 48 h. The results indicated that TGF-β1 and PA3611 increased the expression of α-SMA and vimentin and decreased the expression of *E*-cadherin and ZO-1 compared with PBS, while PA3611 + TGF-β1-neutralizing antibody significantly decreased the expression of α-SMA and vimentin and increased the expression of E-cadherin and ZO-1; the change trends in the PA3611 + TGF-β1-neutralizing antibody group were similar to those in the PBS group (Figures 2C–G). These results indicated that PA3611-promoted bronchial epithelial cell EMT was regulated by TGF-β1.

Many studies have revealed that NF-κB and p65 phosphorylation play a key role in the EMT process (Ma et al., 2016; Huang et al., 2018; Zhang et al., 2019; Liu J. et al., 2020). To clarify the effect of PA3611 on the expression of p65, the level of p-p65 and the EMT process, 16HBE14o- and RTE cells were treated with PBS (negative control), TGF-β1 (2 ng/mL, positive control), PA3611 (10 μg/mL), and PA3611 (10 μg/mL) + TGF-β1-neutralizing antibody (10 μg/mL) for 48 h. The results indicated that TGF-β1 and PA3611 increased the expression of p-p65, while PA3611 + TGF-β1-neutralizing antibody significantly decreased the expression of p-p65 (Figures 2H,I). These results indicated that PA3611 promoted EMT by stimulating TGF-β1-induced phosphorylation of p65.

### p65 Upregulated Epithelial-Mesenchymal Transition-Related Protein Expression Without Affecting the Level of TGF-β1 and p38 Phosphorylation

**FIGURE 3:**
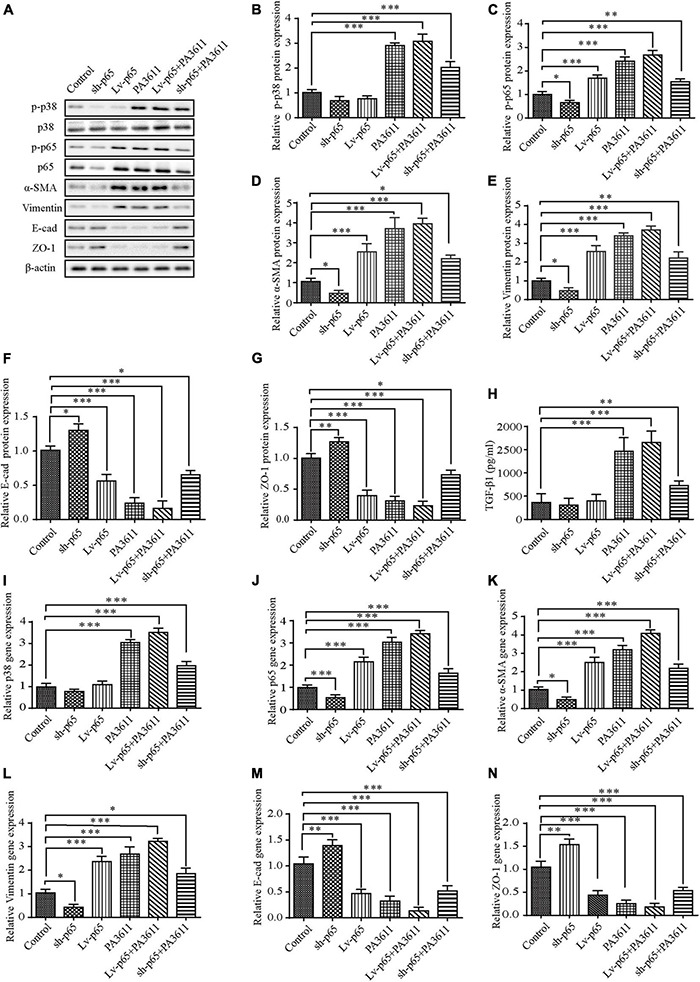
Effects of p65 overexpression or knockdown on the expression of TGF-β1, p38 phosphorylation and the expression of EMT-related markers. 16HBE14o- and RTE cells were transfected with a control vector (indicated as blank), a p65 overexpression vector (indicated as p65), or a specific siRNA directed against p65 (indicated as sh-p65). Twenty-four hours post-transfection, cells were treated with PBS (negative control) or PA3611 (10 μg/mL) for another 48 h. Then, cells were collected to examine the gene and protein expression of p38, p65 and EMT-related markers **(A–G,I–N)**. Cell culture supernatants were collected for TGF-β1 level evaluation by enzyme-linked immunosorbent assay **(H)**. The results are representative of three independent experiments. The data are presented as the mean ± SD. *P < 0.05, ^**^P < 0.01, ^***^P < 0.001, vs. the control group.

To verify the effect of p65 on the EMT process and the expression of related pathway factors, p65 was overexpressed in 16HBE14o- and RTE cells via transfection with pcDNA3.1/p65 cDNA or knocked down through transfection with a specific siRNA directed against p65, and the cells were then treated with PBS or PA3611 (10 μg/mL) for 48 h. The results showed that both p65 overexpression and PA3611 treatment upregulated p65 phosphorylation and α-SMA and vimentin expression and decreased the levels of *E*-cadherin and ZO-1 at the gene and protein levels; moreover, the expression levels changed more significantly with the combination of treatment with PA3611 and p65 overexpression (Figures 3A,C–G,J–N). However, the levels of TGF-β1 and p38 phosphorylation were not affected by either overexpression or knockdown of p65 (Figures 3A,B,H,I). After the addition of PA3611, TGF-β1 levels and p38 expression were significantly upregulated regardless of the p65 expression status (Figures 3A,B,H,I). These results indicated that PA3611 has a regulatory effect on the secretion of TGF-β1 and the phosphorylation of p38 and p65 and that PA3611 promotes bronchial epithelial cell EMT by affecting the secretion of TGF-β1 and regulating p65 phosphorylation.

### PA3611 but Not TGF-β1 Stimulated avβ6 Expression

**FIGURE 4:**
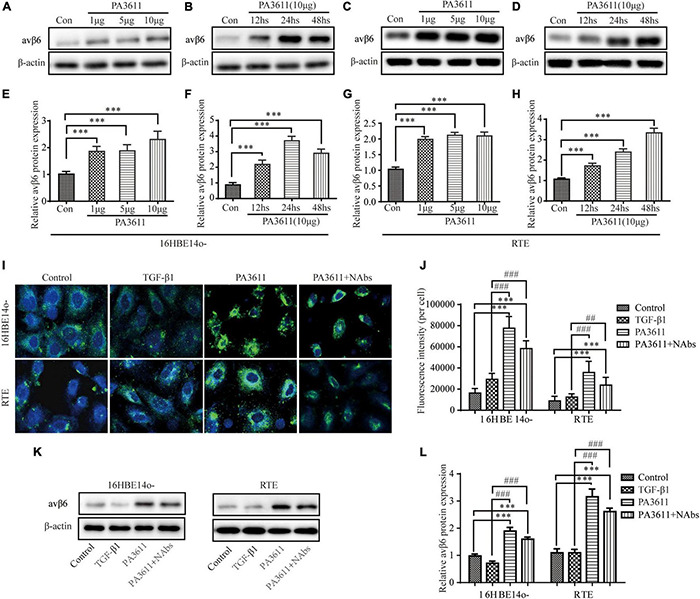
PA3611 but not TGF-β1 stimulated avβ6 protein expression. 16HBE14o- and RTE cells were treated with different concentrations of PA3611 (1, 5, and 10 μg/mL) for different durations (12, 24, and 48 h), and αvβ6 expression was detected by Western blotting **(A–H)**. 16HBE14o- and RTE cells were treated with PBS, TGF-β1 (2 ng/mL), PA3611 (10 μg/mL), and PA3611 (10 μg/mL) + TGF-β1-neutralizing antibody (10 μg/mL) for 48 h, and αvβ6 expression was detected by immunofluorescence staining **(I,J)** and Western blot **(K,L)**. The results are representative of three independent experiments. The data are presented as the mean ± SD. *P < 0.05, ^**^P < 0.01, ^***^P < 0.001, vs. the control group; #*P* < 0.05, ##*P* < 0.01, ###*P* < 0.001, vs. the TGF-β1 group.

Integrin αvβ6, as a transmembrane protein, is the only member of the integrin family expressed in epithelial cells. It is expressed at very low levels in normal epithelial cells, but its expression increases significantly in response to injury or inflammatory stimulation (Munger et al., 1999). Integrin αvβ6 can activate endogenous TGF-β1 in a paracrine manner and is involved in the regulation of its signaling pathway (Puthawala et al., 2008; Katsumoto et al., 2011). To determine whether PA3611 stimulation can increase the expression of αvβ6, 16HBE14o- and RTE cells were treated with different concentrations of PA3611 (1, 5, and 10 μg/mL), and αvβ6 expression was detected after different durations of treatment (12, 24, and 48 h). The results showed that PA3611 promoted αvβ6 expression in a concentration- and time-dependent manner; with an increase in PA3611 concentration and prolongation of the stimulation time, the expression of αvβ6 increased gradually (Figures 4A–H). To clarify whether PA3611 or TGF-β1 affects the expression of αvβ6, 16HBE14o- and RTE cells were treated with PBS, TGF-β1 (2 ng/mL), PA3611 (10 μg/mL), and PA3611 (10 μg/mL) + TGF-β1-neutralizing antibody (10 μg/mL) for 48 h, and αvβ6 expression was detected by immunofluorescence staining (Figures 4I,J) and Western blot (Figures 4K,L). The results indicated that PA3611 but not TGF-β1 stimulated avβ6 expression. Even after the addition of TGF-β1-neutralizing antibody, the level of αvβ6 was still higher than that in the control or TGF-β1 group (Figures 4I–L).

### PA3611 Stimulation of TGF-β1 Expression and Induction of Epithelial-Mesenchymal Transition Could Be Inhibited by Blocking Integrin αvβ6

**FIGURE 5:**
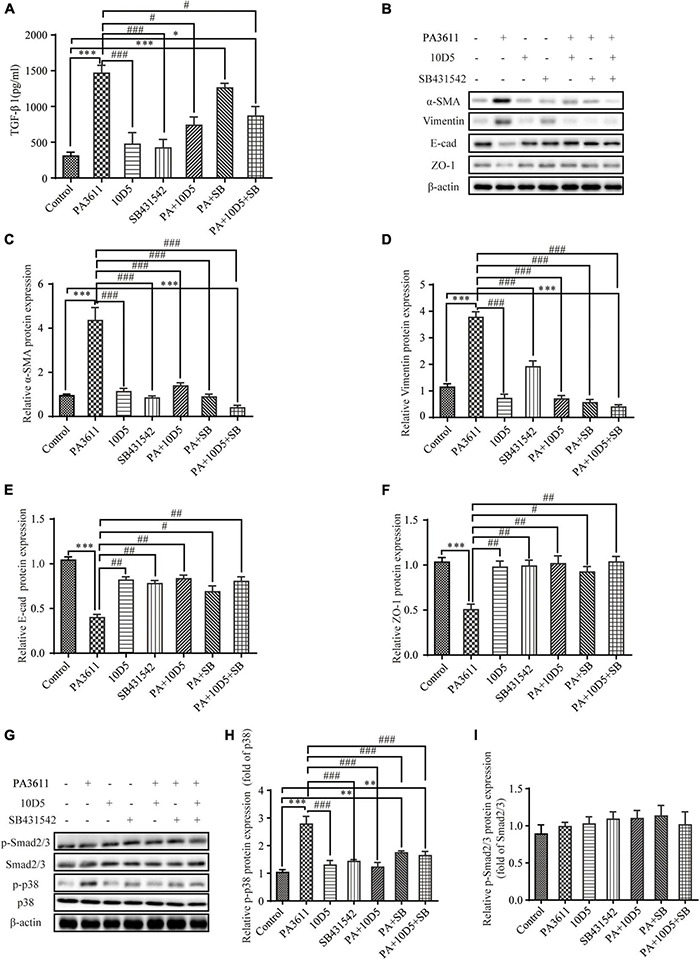
PA3611 stimulated TGF-β1 expression and induced EMT, which could be inhibited by blocking integrin αvβ6. 16HBE14o- cells were pretreated with the integrin αvβ6-blocking antibody 10D5 (10 μM) or a specific inhibitor of TGF-β1-Smad2/3 signaling, SB431542 (10 μM), for 2 h prior to incubation with PA3611 (10 μg/mL) for 48 h. The TGF-β1 levels in cell culture supernatants were evaluated by ELISA **(A)**. EMT-related proteins **(B–F)** and signaling pathway proteins **(G–I)** were detected using Western blotting. The results are representative of three independent experiments, and the data are presented as the means ± SD. *P < 0.05, ^**^P < 0.01, ^***^P < 0.001, vs. the control group; #*P* < 0.05, ##*P* < 0.01, ###*P* < 0.001, vs. the PA3611 group.

To further investigate whether PA3611 stimulated TGF-β1 expression and induced EMT through the regulation of αvβ6, 16HBE14o- cells were pretreated with the integrin αvβ6-blocking antibody 10D5 (10 μM) or a specific inhibitor of TGF-β1-Smad2/3 signaling, SB431542 (10 μM), for 2 h prior to incubation with PA3611 (10 μg/mL) for 48 h. The TGF-β1 levels in cell culture supernatants were evaluated by ELISA, and EMT-related proteins were detected using Western blotting. The results showed that the integrin αvβ6-blocking antibody 10D5 significantly inhibited PA3611-induced TGF-β1 secretion, whereas the specific inhibitor of TGF-β1 did not have a similar effect (Figure 5A). Furthermore, the PA3611-induced increase in the expression of α-SMA and vimentin was significantly attenuated by the integrin-αvβ6 blocking antibody 10D5, and the decreased expression of E-cadherin and ZO-1 was attenuated (Figures 5B–F). These results indicated that integrin αvβ6 played a critical role in the PA3611-induced EMT process. However, the role of the TGF-β1-specific inhibitor SB431542 in the PA3611-induced EMT process was not significant, suggesting that PA3611-induced EMT in epithelial cells may be mediated by pathways other than the Smad2/3 signaling pathway (Figures 5B–F).

### Integrin αvβ6 Mediated PA3611-Induced Epithelial-Mesenchymal Transition via the TGF-β1-p38 Signaling Pathway

Previous studies have shown that the activity of TGF-β1 is not exclusive to the SMAD signaling pathway; it can also regulate and be regulated by other signaling pathways. For example, TGF-β1 has been shown to activate extracellular signal-regulated kinases (ERKs), p38, c-Jun N-terminal kinases (JNKs), and mitogen-activated protein kinases (MAPKs) (Derynck and Zhang, 2003; Moustakas and Heldin, 2005). To verify whether PA3611 promotes EMT through αvβ6-mediated TGF-β1 regulation of p38 phosphorylation, 16HBE14o- cells were pretreated with the integrin αvβ6-blocking antibody 10D5 (10 μM) or a specific inhibitor of TGF-β1-Smad2/3 signaling, SB431542 (10 μM), for 2 h prior to incubation with PA3611 (10 μg/mL) for 48 h. Smad2/3 and p38 proteins were detected using Western blot analysis. The results showed that PA3611 significantly increased the level of p38 phosphorylation, and the integrin αvβ6-blocking antibody 10D5 inhibited the increase in the level of p-p38, as did the TGF-β1-specific inhibitor SB431542. However, no similar changes were observed in the phosphorylation levels of smad2/3 (Figures 5G–I). These results indicated that PA3611 promoted EMT through the non-Smad signaling pathway.

### p38 Regulated p65 Phosphorylation and Epithelial-Mesenchymal Transition-Related Protein Expression Without Affecting TGF-β1

**FIGURE 6:**
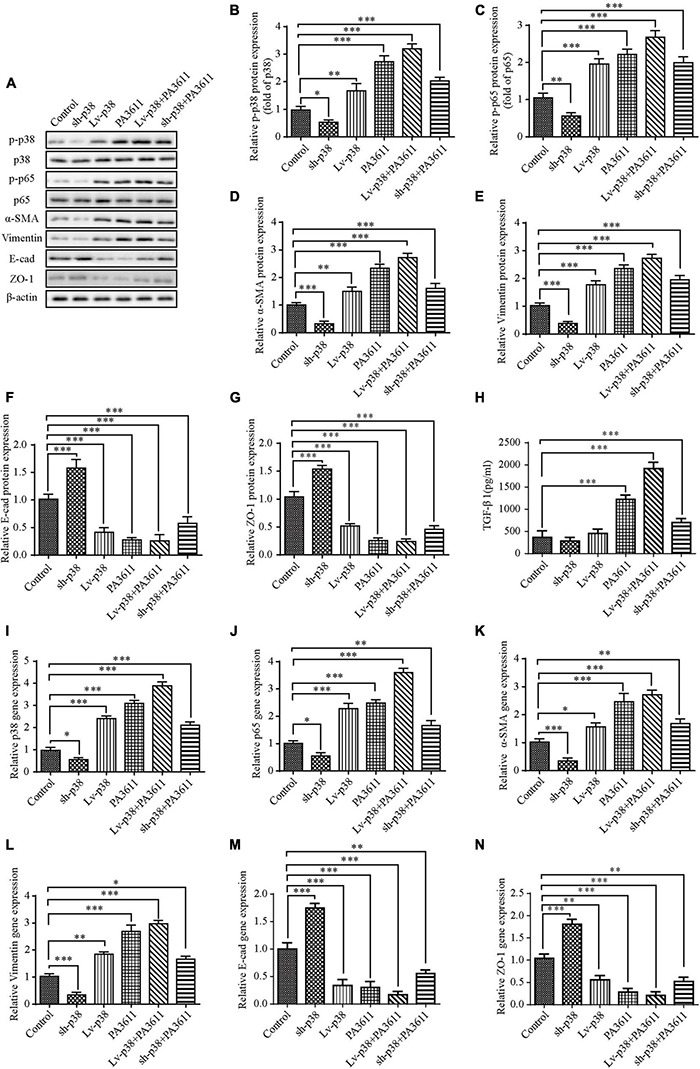
Effects of p38 overexpression or knockdown on the expression of TGF-β1, EMT-related marker expression and the phosphorylation of p38 and p65. 16HBE14o- cells were transfected with a control vector (indicated as blank), a p38 overexpression vector (indicated as p38), or a specific siRNA directed against p38 (indicated as sh-p38). Twenty-four hours post-transfection, cells were treated with PBS (negative control) or PA3611 (10 μg/mL) for another 48 h. Then, cell culture supernatants were collected for TGF-β1 level evaluation by enzyme-linked immunosorbent assay **(H)**, and cells were collected to examine the gene and protein expression of p38, p65 and EMT-related markers **(A–G,I–N)**. The results are representative of three independent experiments. The data are presented as the means ± SD. *P < 0.05, ^**^P < 0.01, ^***^P < 0.001, vs. the control group.

To further identify the function of p38 in PA3611-induced bronchial epithelial cell EMT, 16HBE14o- cells were transfected with pcDNA3.1/p38 cDNA or a specific siRNA directed against p38 and then treated with PBS or PA3611 (10 μg/mL) for 48 h. p38, p65 and EMT-related gene and protein levels were assessed by real-time PCR and Western blot analysis. The results showed that overexpression of p38 resulted in upregulation of p65 phosphorylation and p65 gene expression (Figures 6A,C,J); and p38 overexpression resulted in activation of p38 phosphorylation and p38 gene expression (Figures 6A,B,I) moreover, p38 overexpression upregulated the expression of α-SMA and vimentin and decreased the levels of *E*-cadherin and ZO-1 at the gene and protein levels (Figures 6A,D–G,K–N).

Additionally, the expression levels changed more significantly with the combination of treatment with PA3611 and p38 overexpression (Figures 6A,C–N), and with knockdown of p38, the results were the opposite of those obtained by p38 overexpression (Figures 6A,C–N); however, neither overexpression nor knockdown of p38 affected the secretion of TGF-β1. In contrast, TGF-β1 expression significantly increased with the combination of treatment with PA3611 and overexpression or knockdown of p38 (Figure 6H). These data indicated that PA3611 promoted bronchial epithelial cell EMT via the TGF-β1-p38 signaling pathway.

### PA3611 Promoted Bronchial Epithelial Cell Epithelial-Mesenchymal Transition *in vivo*

**FIGURE 7:**
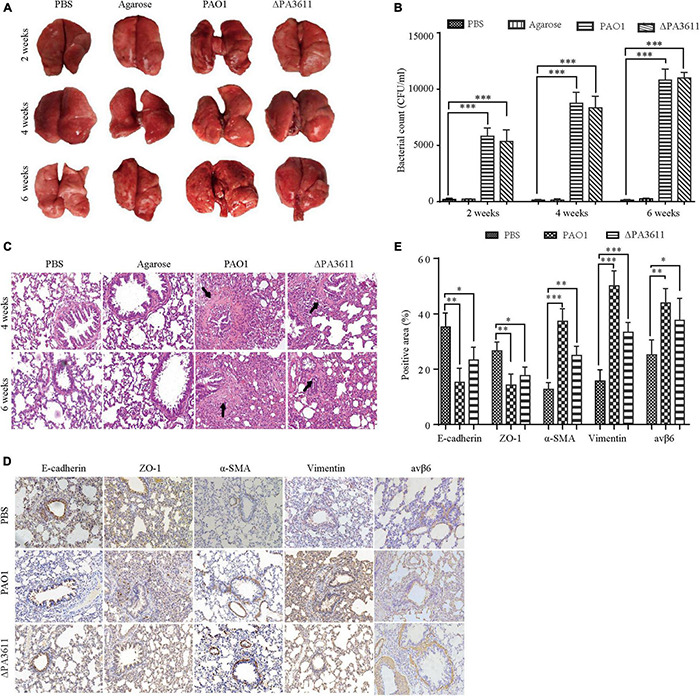
Effects of PA3611 on lung infection and EMT *in vivo*. Six- to eight-week-old male Wistar rats were intratracheally infected with the PAO1 or ΔPA3611 bacterial strain. The strains were coated with agarose and agarose, and PBS was used as a control. Lungs were collected at 2, 4, or 6 weeks post-infection. Overall gross views of lung tissues from the four groups at 2, 4, and 6 weeks post-infection are shown **(A)**. Bacterial load tests were performed in the above four groups at 2, 4, and 6 weeks post-infection **(B)**. HE staining **(C)** and immunohistochemical staining of *E*-cadherin, ZO-1, α-SMA, vimentin and integrin αvβ6 **(D)** were performed. The scale bar represents 100 μm. Morphometric analysis of EMT-related protein-positive areas and integrin αvβ6 was performed on immunohistochemically stained sections of lung tissues **(E)**. The lung injury results are representative of three independent experiments. The data are presented as the mean ± SD, NS, no significance, *P < 0.05, ^**^P < 0.01, ^***^P < 0.001, vs. the control group.

To verify the function of PA3611 in PA infection and EMT *in vivo*, 6- to 8-week-old male Wistar rats were intratracheally infected with PBS, agarose, agarose-coated PAO1 or agarose-coated ΔPA3611. The lung morphology results showed mild local hyperemia in the agarose-coated PAO1 group at 2 weeks postinfection but not in the other groups. At 4 weeks postinfection, scattered nodules on the lung surface with local bleeding points were seen in the agarose-coated PAO1 and agarose-coated ΔPA3611 groups, although they were more obvious in the agarose-coated PAO1 group. These phenomena were more pronounced at 6 weeks postinfection (Figure 7A). The CFU assessment results showed that there was no significant difference between the agarose-coated PAO1 group and agarose-coated ΔPA3611 group at 2, 4, or 6 weeks post-infection, which demonstrated that the decrease in bronchial epithelial cell EMT may be attributed to the decrease in PA strain virulence due to PA3611 deletion (Figure 7B). HE staining revealed alveolar cavity collapse, intra-alveolar hemorrhage, partial pulmonary septum rupture, alveolar septum widening, bronchial lumen stenosis and deformation, smooth muscle proliferation and fibrosis around the trachea, and inflammatory cell infiltration in the lung interstitium in the agarose-coated PAO1 and ΔPA3611 groups starting at 4 weeks postinfection, and these changes were more obvious in the agarose-coated PAO1 group and were more pronounced at 6 weeks postinfection (Figure 7C). Immunohistochemistry analyses of EMT-related markers and integrin αvβ6 confirmed that rats treated with agarose-coated PAO1 and agarose-coated ΔPA3611 exhibited increased α-SMA, vimentin and integrin αvβ6 protein expression and decreased expression of *E*-cadherin and ZO-1 (Figures 7D,E), which was consistent with the *in vitro* results and indicated that PA3611 played a role in causing lung infection and promoting bronchial epithelial cell EMT.

## Discussion

*Pseudomonas aeruginosa* is one of the main pathogens of chronic airway diseases, such as COPD, bronchiectasis, and bronchiolitis obliterans, associated with chronic inflammation and structural changes (Moore and Mastoridis, 2017). Although the specific mechanism by which PA infection causes related airway remodeling is still unclear, it is clear that EMT plays a key role in the process of bronchial fibrosis (Nowrin et al., 2014). In this study, we demonstrated that PA3611 could play a crucial role in PA-associated airway remodeling via integrin αvβ6-mediated TGFβ1-induced p38/NF-κB pathway activation.

In this study, a recombinant PA3611 protein was constructed through prokaryotic expression, and this protein had a tendency to induce EMT in bronchial epithelial cells. We first studied the effect of the protein on the proliferation of 16HBE14o- and RTE cell lines, and the results showed that PA3611 could inhibit cell proliferation in a time- and dose-dependent manner.

The TEM results showed that after PA3611 stimulation of the above cells for 48 h, in addition to enlargement of the intercellular space and disappearance of intercellular connections, filamentous pseudopodia appeared, and the ultrastructural manifestations of nuclear abnormity, perinuclear space broadening, mitochondrial swelling and distortion, and intracellular glycogen were exacerbated, consistent with the ultrastructural changes previously reported to occur with EMT (Ranchoux et al., 2015; Gagliano et al., 2016; Wang et al., 2017). The possible correlation between inhibition of cell proliferation and EMT has not been confirmed to date; however, our study revealed that PA3611 significantly promoted the occurrence of EMT while also inhibiting cell proliferation.

Epithelial-mesenchymal transition is a pathological process in which cells lose their original epithelial cell polarity and transform into cells with mesenchymal cell morphology and characteristics. The expression of original epithelial cell markers, such as *E*-cadherin and ZO-1, was decreased, and the expression of mesenchymal cell markers, such as α-SMA, N-cad and vimentin, was increased. There are currently some controversies regarding α-SMA as a marker for mesenchymal cells; for example, in skeletal muscle fibrosis, α-SMA is not a marker of fibrogenic cell activity (Zhao et al., 2018), and α-SMA is an inconsistent marker of fibroblasts responsible for force-dependent TGF-β activation or collagen production across multiple models of organ fibrosis (Sun et al., 2016). However, this is mainly in contracting fibroblasts, collagen-producing fibroblasts and skeletal muscle fibrosis. In this study, we mainly analyzed the EMT of bronchial epithelial cells, and α-SMA was still used as a marker of mesenchymal cells. We detected the expression of EMT-related proteins in 16HBE14o- and RTE cells stimulated with PA3611 protein for 24, 48, or 72 h and found that the expression of the epithelial cell markers *E*-cadherin and ZO-1 decreased, while that of the mesenchymal cell markers vimentin and α-SMA increased, indicating that the cells had undergone EMT-related changes.

TGF-β1 has been considered a key inducer of EMT and has a central role in regulating fibrosis through several different mechanisms (Kalluri and Neilson, 2003; O’Connor and Gomez, 2014; Dongre and Weinberg, 2019). Studies have also shown that the nuclear transcription factor NF-κB can regulate the process of EMT by controlling the Smad-independent gene network (Tian et al., 2015). In addition, reducing the activation of NF-κB can alleviate alveolar epithelial cell EMT induced by bleomycin, and inhibiting NF-κB can prevent bronchial epithelial cell EMT induced by tumor necrosis factor ligand superfamily member 14 (TNFSF14) (Hung et al., 2015; Zhao et al., 2015). In this study, the intracellular NF-κB p65 phosphorylation levels in the group treated with PA3611 stimulation for 48 h were significantly higher than those in the control group. The TGF-β1-neutralizing antibody group showed increased expression of *E*-cadherin and ZO-1 and decreased expression of vimentin and α-SMA compared with the PA3611- and TGF-β1-stimulated groups, consistent with the expression levels in the control group, and the phosphorylation level of NF-κB p65 was reduced in the TGF-β1-neutralizing antibody group. These results indicated that blocking TGF-β1 could inhibit the occurrence of PA3611-induced EMT, suggesting that PA3611-induced EMT changes in epithelial cells were achieved by stimulating TGF-β1 secretion.

Integrin αvβ6 is a member of the integrin family and is exclusively expressed in epithelial cells, where it can serve as a fibronectin receptor (Koivisto et al., 2018). It has been found that the expression of integrin αvβ6 is extremely low in normal airway epithelial cells, alveolar epithelial cells, and renal and skin epithelial cells and is significantly increased upon injury, inflammation, or epithelial tumor development (Sheppard, 2003). As an activator of TGF-β (Munger et al., 1999; Shi et al., 2011; John et al., 2020), integrin αvβ6 participates in the regulation of a wide array of cell behaviors, including cell proliferation, migration, and survival; tissue invasion; and innate immunity (Hynes, 2002; Campbell and Humphries, 2011). In our study, we found that the expression of integrin αvβ6 was significantly increased in 16HBE14o- and RTE cells stimulated with PA3611, and the change became more significant with increasing concentration and prolongation of stimulation time, which is consistent with the study by Breuss et al. (1995) on the expression of integrin αvβ6 in airway epithelium in the PA-infected lower respiratory tract. This finding suggests that integrin αvβ6 may be involved in the regulation of bronchial epithelial cell EMT.

To further investigate the PA3611-mediated role of integrin αvβ6 in bronchial epithelial cell EMT and the related mechanisms, the integrin αvβ6-blocking antibody 10D5 and the TGF-β1-specific inhibitor SB431542 were used to treat 16HBE14o- and RTE cells; subsequently, TGF-β1 expression, EMT-related protein levels and the levels of proteins in related signaling pathways were detected. We found that the integrin αvβ6-blocking antibody 10D5 but not the TGF-β1-specific inhibitor SB431542 significantly inhibited PA3611-induced TGF-β1 secretion. Furthermore, the PA3611-induced increase in the expression of α-SMA and vimentin was significantly attenuated by the integrin αvβ6-blocking antibody 10D5, while the decrease in the expression of *E*-cadherin and ZO-1 was attenuated. PA3611 significantly increased the level of p38 phosphorylation, and the integrin αvβ6-blocking antibody 10D5 inhibited the increase in the level of p-p38, as did the TGF-β1-specific inhibitor SB431542. However, no similar changes in smad2/3 phosphorylation levels were observed. These results indicated that integrin αvβ6 played a critical role in the PA3611-induced EMT process that was mediated by the TGF-β1-induced p38 pathway rather than the Smad2/3 signaling pathway.

Although canonical TGF-β signaling via TGF-β type I receptor and intracellular Smad2/3 proteins has received considerable attention (Mackinnon et al., 2012; Thien et al., 2015; Alyaseer et al., 2020), emerging data suggest that aberrant activation of non-canonical TGF-β signaling via non-Smad2/3 pathways plays a key role in the pathogenesis of fibrosis (Finnson et al., 2020). In contrast to the findings of Liu W. et al. (2020), who reported that integrin αvβ6 regulated BEAS-2B cell EMT by affecting the LPS-activated TGF-β1-Smad2/3 signaling pathway, we found that PA3611 regulated epithelial cell EMT via integrin αvβ6-mediated TGF-β1-p38 signaling pathway activation, and our differing results may be related to the different stimuli, cell types, and stimulation times used in each study.

In this study, we found that PA3611 could promote the occurrence of EMT in epithelial cells by mediating integrin αvβ6. However, other functions of PA3611 need to be elucidated in further research. Some questions include the following: What can simulate PA to secrete sufficient amounts of PA3611? How high is the level of PA3611 in the airway lining fluid, and can it be detected in bronchoalveolar lavage? Is PA3611 detectable in the blood if the lung epithelium becomes leaky due to inflammation? What mechanism drives the posttranscriptional modification of secreted PA3611 to enhance PA virulence? In addition, considering the high prevalence of fibrotic lung diseases in men, is PA3611 abnormally expressed in these patients? These questions are expected to be answered in further research on PA3611.

According to our results, we conclude that PA infects bronchial epithelial cells by secreting the PA3611 protein, which promotes EMT via integrin αvβ6-mediated TGF-β1-induced p38/NF-κB pathway activation, and we believe that these results provide a new potential target for the clinical treatment of bronchial EMT and fibrosis caused by chronic PA infection.

## Data Availability Statement

The original contributions presented in the study are included in the article/Supplementary Material, further inquiries can be directed to the corresponding author/s.

## Ethics Statement

The animal study was reviewed and approved by the Institutional Animal Ethics Committee of the Second Affiliated Hospital of Nanjing Medical University.

## Author Contributions

LS, SC, and GF conceived and designed the study, conducted the research, and revised the manuscript. SL, HL, YS, JY, LQ, YZ, XL, KD, XC, and XD analyzed and interpreted the data. LS, SL, and GF wrote the manuscript. All authors read and approved the final version of the manuscript.

## Conflict of Interest

The authors declare that the research was conducted in the absence of any commercial or financial relationships that could be construed as a potential conflict of interest.

## Publisher’s Note

All claims expressed in this article are solely those of the authors and do not necessarily represent those of their affiliated organizations, or those of the publisher, the editors and the reviewers. Any product that may be evaluated in this article, or claim that may be made by its manufacturer, is not guaranteed or endorsed by the publisher.
