# Supplementary material for: The Pseudomonas aeruginosa Secreted Protein PA3611 Promotes Bronchial Epithelial Cell Epithelial-Mesenchymal Transition via Integrin αvβ6-Mediated TGF-β1-Induced p38/NF-κB Pathway Activation
Source: Front Microbiol. 2022 Feb 7;12:763749. doi: 10.3389/fmicb.2021.763749 (PMC8860233; doi:10.3389/fmicb.2021.763749)
Supplement: Supplementary file 1 [file Data_Sheet_1.docx]

**Supporting information**

**S1 Table A list of primers used for PCR**

| **Primer** | **Sequence** |
| --- | --- |
| PA3611-Forward: | 5′-GATGGATCCGCGAGCCTGAAAGACTTCG3′ |
| PA3611-Reverse: | 5′-GACCTCGAGTTATTTTTTGCCCTGAATGC-3′ |
| Human-p65-Forward: | 5’-CTGTCCTTTCTCATCCCATCTT-3’ |
| Human-p65-Reverse: | 5’-TCCTCTTTCTGCACCTTGTC-3’ |
| Human-p38-Forward: | 5’-CGTGTTGCAGATCCAGACCA-3’ |
| Human-p38-Reverse: | 5’-GCCAGA ATGCAGCCTACAGA-3’ |
| Human-α-SMA-Forward: | 5’-CCGACCGAATGCAGAAGGA-3’ |
| Human-α-SMA-Reverse: | 5’-ACAGAGTATTTGCGCTCCGAA-3’ |
| Human-Vimentin-Forward: | 5’-GTTTCCCCTAAACCGCTAGG-3’ |
| Human-Vimentin-Reverse: | 5’-AGCGAGAGTGGCAGAGGA-3’ |
| Human-E-cadherin-Forward: | 5’- GACCGGTGCAATCTTCAAA-3’ |
| Human-E-cadherin- Reverse: | 5’- TTGACGCCGAGAGCTACAC-3’ |
| Human-ZO-1-Forward: | 5’- GTGCCAGGAAGTTATACGAGCG-3’ |
| Human-ZO-1- Reverse: | 5’- CACCATACCAACCATCATTCATTG-3’ |
| Human-GAPDH-Forward: | 5’-CAGGGCTGCTTTTAACTCTGGTAA-3’ |
| Human-GAPDH-Reverse: | 5’-GGGTGGAATCATATTGGAACATGT-3’ |
| Rat-p65-Forward: | 5’-AACAACACAGACCCAGGAGT-3’ |
| Rat-p65-Reverse: | 5’-CTGTCACCAGGCGAGTTATAG-3’ |
| Rat-p38-Forward: | 5’-GTGATTGGTCTGTTGGATGTG-3’ |
| Rat-p38-Reverse: | 5’-TGGATTATGTCAGCCGAGTG-3’ |
| Rat-α-SMA-Forward: | 5’-AGAAGCCCAGCCAGTCGCCATCA-3’ |
| Rat-α-SMA-Reverse: | 5’-AGCAAAGCCCGCCTTACAGAGCC-3’ |
| Rat-Vimentin-Forward: | 5’-CCCAGATTCAGGAACAGCAT-3’ |
| Rat-Vimentin-Reverse: | 5’-CACCTGTCTCCGGTATTCGT-3’ |
| Rat-E-cadherin-Forward: | 5’-AAGACCAACGAGGGCATTC-3’ |
| Rat-E-cadherin- Reverse: | 5’-GCTCTCGCGCAGTGTAAGAT-3’ |
| Rat-ZO-1-Forward: | 5’-AGCGAAGCCACCTGAAGATA-3’ |
| Rat-ZO-1- Reverse: | 5’-GATGGCCAGCAGGAATATGT-3’ |
| Rat-GAPDH-Forward: | 5’- CCTTCATTGACCTCAACTACATG-3’ |
| Rat-GAPDH-Reverse: | 5’- CCTTCTCCATGGTGGTGAAGAC-3’ |

**Tab S1:** The Relevant primer sequences used in PCR experiments in this study,

**S2 Table A list of RNA for transfection**

| **RNA** | **Sequence** |
| --- | --- |
| p65-Homo-specific siRNA | 5’-GCCCUAUCCCUUUACGUCATT-3’ |
| p65-Rat-specific siRNA | 5’-AGUCCCUGUCUGCACCUGUTT-3’ |
| p38-Homo-specific siRNA | 5’-GCAUAAUGGCCGAGCUGUUTT-3’ |
| p38-Rat-specific siRNA | 5’-GACUGUGAGCUCAAGAUUCTT-3’ |
| A non-specific siRNA | 5’-UUCUCCGAAGGUGUCACGUTT-3’ |
| a NC miRNA for inhibitor | 5’-CAGUACUUUUGUGUAGUACAA-3’ |

Abbreviation: NC: negative control

**Table S2:** Related primers used in RNA transfection. NC means negative control.

**
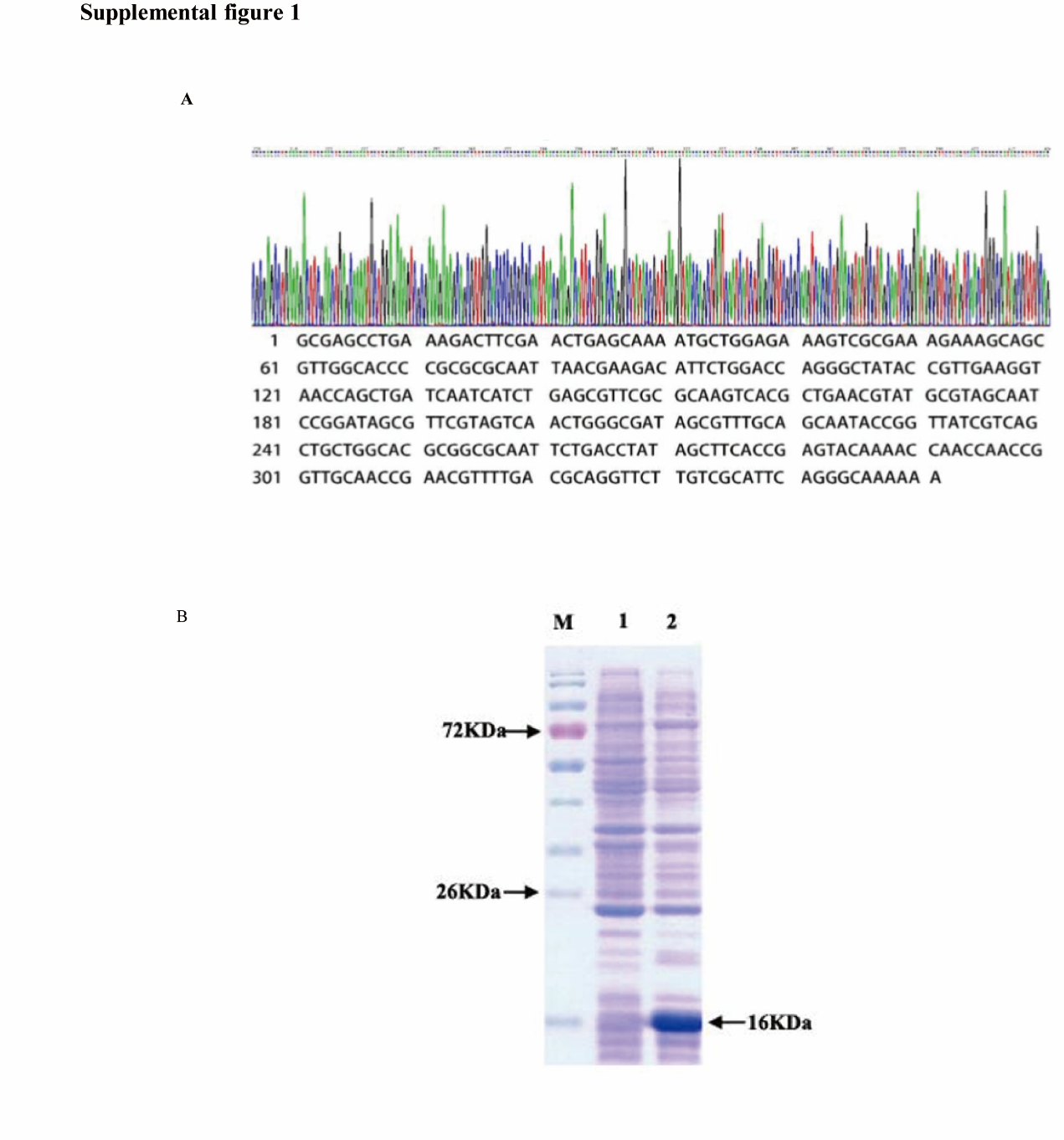
**

**Supplemental figure 1:** Heterologous expression and puriﬁcation of recombinant PA3611.Sequencing results of the PET-28a-PA3611 recombinant plasmid (Suppl Fig. 1A). Expression, purification and identification of the PA3611 protein (M: molecular weight marker of the protein; 1: total protein of the bacteria without induction; 2: total protein of the induced bacteria; Suppl Fig. 1B).
